# Ginkgolide B Attenuates 6-Hydroxydopamine-Induced Cellular Damage and Modulates Oxidant-Evoked Ca^2+^ Signalling in SH-SY5Y Cells

**DOI:** 10.3390/cimb48090945

**Published:** 2026-09-16

**Authors:** Ramazan Çınar, Merve Göztepe, Adem Ahlatcı, Kenan Yıldızhan

**Affiliations:** 1 Department of Biophysics, Faculty of Medicine, Bitlis Eren University, 13100 Bitlis, Türkiye; rcinar@beu.edu.tr; 2 Department of Medical Services and Techniques, Vocational School of Health Services, Bilecik Seyh Edebali University, 11230 Bilecik, Türkiye; merve.celen@bilecik.edu.tr; 3 Vocational School of Health Services, Van Yuzuncu Yil University, 65080 Van, Türkiye; ademahlatci@gmail.com; 4 Department of Biophysics, Faculty of Medicine, Van Yuzuncu Yil University, 65080 Van, Türkiye

**Keywords:** Ginkgolide B, 6-hydroxydopamine, SH-SY5Y, oxidative stress, intracellular Ca^2+^, cell death, Parkinson’s disease

## Abstract

Oxidative stress and disruption of intracellular Ca^2+^ homeostasis contributes to dopaminergic neuronal damage in Parkinson’s disease. In this study, the protective effects of Ginkgolide B (GKB) against 6-hydroxydopamine (6-OHDA)-induced SH-SY5Y cell damage were investigated, with particular attention to oxidant-evoked intracellular Ca^2+^ signalling. Cells were pre-treated with GKB (20 µg/mL) for 2 h, followed by exposure to 6-OHDA (200 µM) for 24 h. CCK-8-derived metabolic activity, oxidative stress, cell death, PARP-1, caspase-3, and intracellular Ca^2+^ responses were evaluated using biochemical and fluorescence methods. 6-OHDA decreased CCK-8-derived metabolic activity, GSH, and SOD levels, while increasing MDA, intracellular oxidant-sensitive fluorescence, PI-positive cell ratio, and total PARP-1 and caspase-3 levels. GKB pretreatment significantly attenuated these changes and partially improved cellular redox balance. Furthermore, while 6-OHDA enhanced the H_2_O_2_-stimulated intracellular Ca^2+^ response, GKB reduced this increase. ACA attenuated the H_2_O_2_-evoked Ca^2+^ signal, indicating the involvement of an ACA-sensitive Ca^2+^-entry component. However, because ACA is not selective for TRPM2, these pharmacological findings cannot establish a TRPM2-dependent mechanism. The findings demonstrate that GKB reduces 6-OHDA-induced SH-SY5Y cell damage by supporting antioxidant defence, limiting cell death, and attenuating the enhanced oxidant-stimulated Ca^2+^ response.

## 1. Introduction

Parkinson’s disease (PD) is a chronic neurodegenerative disease characterized primarily by the progressive loss of dopaminergic neurons in the substantia nigra pars compacta region and a decrease in striatal dopamine. Bradykinesia, rigidity, resting tremor, and postural instability are the main motor findings, while the disease also presents with a broad clinical picture, including cognitive, autonomic, sensory, and neuropsychiatric impairments [1,2]. Although current treatments are effective in controlling symptoms, they cannot completely halt dopaminergic neurodegeneration. Therefore, elucidating the cellular mechanisms that initiate and propagate neuronal damage and developing new neuroprotective approaches remains crucial.

The pathogenesis of PD is a multifactorial process involving interactions among mitochondrial dysfunction, α-synuclein aggregation, impaired proteostasis, neuroinflammation, oxidative stress, and alterations in intracellular Ca^2+^ homeostasis [2,3,4]. The formation of reactive quinones and reactive oxygen species (ROS) during dopamine metabolism makes dopaminergic neurons particularly susceptible to oxidative damage. Excessive ROS production can lead to damage to lipids, proteins, and nucleic acids, loss of mitochondrial function, and activation of cell death pathways [5,6]. During this process, antioxidant defence components such as reduced glutathione (GSH) and superoxide dismutase (SOD) decrease, while cellular damage markers such as malondialdehyde (MDA), poly(ADP-ribose) polymerase-1 (PARP-1), and caspase-3 may increase.

6-Hydroxydopamine (6-OHDA) is a neurotoxin widely used to induce dopaminergic neuronal damage in experimental Parkinsonism models [7,8]. Following uptake through catecholamine transporters, 6-OHDA undergoes auto-oxidation and initiates a cascade involving ROS generation, mitochondrial dysfunction, impaired antioxidant defense, and cell death [5,6]. Human-derived SH-SY5Y neuroblastoma cells are frequently used in PD research due to their catecholaminergic properties, suitability for experimental applications, and reproducible response to dopaminergic toxins [9]. However, this model does not fully capture ageing, α-synuclein pathology, neuron–glia interactions, and the disease’s progressive nature. Therefore, 6-OHDA-treated SH-SY5Y cells should be considered a reductionist system for studying specific mechanisms of toxin-induced oxidative neuronal damage, rather than a model that mimics the entirety of PD.

Oxidative stress and intracellular Ca^2+^ dysregulation can mutually reinforce neuronal damage. Increased cytosolic Ca^2+^ increases mitochondrial ROS production and metabolic load, while oxidative stress can also activate redox-sensitive Ca^2+^ channels. Transient Receptor Potential Melastatin 2 (TRPM2) is a non-selective, Ca^2+^-permeable cation channel activated by intracellular ADP-ribose and facilitated by Ca^2+^; oxidative DNA damage can promote channel activation through PARP-1-dependent ADP-ribose generation [10,11]. Long-term activation of TRPM2 can lead to excessive Ca^2+^ influx, mitochondrial dysfunction, and cell death. Increased TRPM2 expression in SH-SY5Y cells has been reported to increase susceptibility to ROS-induced cell death [12,13]. Furthermore, current findings suggest that TRPM2-associated Ca^2+^ signalling under experimental PD conditions can potentiate mitochondrial ROS production and neuronal damage [14,15].

Ginkgolide B (GKB) is a bioactive compound with a diterpene trilactone structure obtained from the *Ginkgo biloba* plant. GKB has been reported to have antioxidant, anti-inflammatory, mitochondrial protective, and anti-apoptotic effects. In SH-SY5Y cells, GKB pre-treatment has been shown to reduce ROS accumulation, protect mitochondrial function, and suppress caspase-dependent cell death [16,17]. Similarly, GKB has been reported to reduce amyloid-β-induced oxidative damage and support GSH and SOD-related antioxidant responses [18]. However, the effects of GKB on 6-OHDA-induced SH-SY5Y cell damage and the relationship of these effects to oxidant-induced Ca^2+^ signalling are not sufficiently elucidated.

In this study, the protective effects of GKB in 6-OHDA-treated SH-SY5Y cells were investigated by assessing CCK-8-derived metabolic activity, oxidative stress, cell death, PARP-1, caspase-3, and intracellular Ca^2+^ responses. The ACA-sensitive component of the H_2_O_2_-evoked Ca^2+^ response was also examined as a pharmacological indicator of redox-sensitive Ca^2+^ entry. ACA has been reported to inhibit TRPM2; however, it also affects other TRP channels, including TRPM8 and TRPC6, and therefore cannot provide TRPM2-specific evidence [13]. Accordingly, the present pharmacological approach was not intended to demonstrate direct TRPM2 activation or a TRPM2-dependent mechanism. We hypothesised that GKB would support cellular redox balance, reduce cell death, and attenuate the enhanced oxidant-evoked Ca^2+^ response under 6-OHDA conditions.

## 2. Materials and Methods

### 2.1. Cell Culture and Chemicals

The human SH-SY5Y neuroblastoma cell line was obtained from the American Type Culture Collection (ATCC, Manassas, VA, USA; catalog no. CRL-2266; Cellosaurus accession: CVCL_0019; RRID: CVCL_0019). The cells were cultured in Dulbecco’s Modified Eagle Medium/Ham’s F-12 (DMEM/F-12) containing 10% heat-inactivated fetal bovine serum (FBS), 100 U/mL penicillin, and 100 µg/mL streptomycin. Cells were maintained at 37 °C in a humidified incubator containing 5% CO_2_ and passaged when approximately 80% confluence was reached. Cell morphology was assessed regularly, and the absence of mycoplasma contamination was confirmed prior to the experiments. 6-Hydroxydopamine hydrobromide (6-OHDA; ≥98% purity by HPLC, containing ascorbic acid as a stabiliser; Cat. No. H116; Sigma-Aldrich, St. Louis, MO, USA) and ginkgolide B (GKB; analytical reference standard, ≥98% purity; Cat. No. G810348; Shanghai Macklin Biochemical Co., Ltd., Shanghai, China) were used in the experiments. Hydrogen peroxide (H_2_O_2_, Cat: 386790-M, Sigma-Aldrich), N-(p-amylcinnamoyl) anthranilic acid (ACA, Cat: A8486, Sigma-Aldrich), dimethyl sulfoxide (DMSO), propidium iodide (PI, Cat: P1304MP, Thermo Fisher Scientific, Waltham, MA, USA), Hoechst (Cat.: 4082, Cell Signaling Technology, Danvers, MA, USA), 2′,7′-dichlorodihydrofluorescein diacetate (DCFH-DA, Cat: C6827, Thermo Fisher Scientific), and Fluo-4 AM (Cat: F14201, Thermo Fisher Scientific) were obtained from the respective sources. GKB and ACA stock solutions were prepared in DMSO and diluted to working concentrations with culture medium immediately before use. The final DMSO concentration was maintained at 0.1% (*v*/*v*) in all experimental groups.

### 2.2. CCK-8 Metabolic Activity Assay

Cellular metabolic activity was evaluated using the Cell Counting Kit-8 (CCK-8; Cat: KTA1020, Abbkine, Wuhan, China) according to the manufacturer’s instructions. The assay is based on the reduction of WST-8 by cellular dehydrogenases via NAD(P)H-dependent activity. Therefore, the CCK-8 signal was interpreted as an indicator of cellular metabolic activity rather than a direct measurement of viable cell number. SH-SY5Y cells were seeded into 96-well plates at 1 × 10^4^ cells per well and incubated for 24 h under standard culture conditions. 6-OHDA was prepared immediately before each experiment in accordance with the manufacturer’s instructions. Briefly, 5 mg of 6-OHDA hydrobromide was reconstituted in 2 mL of sterile phosphate-buffered saline to obtain a 10 mM stock solution containing 0.01% (*w*/*v*) ascorbic acid. Because of its susceptibility to oxidation, the solution was protected from light, kept on ice, and used immediately without storage. The stock solution was diluted 1:50 in pre-warmed complete culture medium to obtain a final 6-OHDA concentration of 200 µM. GKB was dissolved in dimethyl sulfoxide (DMSO) to prepare a 40 mg/mL stock solution. The stock solution was divided into single-use aliquots, protected from light, and stored at −20 °C. Immediately before each experiment, GKB was diluted in complete culture medium to final concentrations of 5, 10, 20, 30, or 40 µg/mL. Based on the molecular weight of GKB (424.4 g/mol), these concentrations corresponded to approximately 11.8, 23.6, 47.1, 70.7, and 94.3 µM, respectively. The final DMSO concentration was adjusted to 0.1% (*v*/*v*) in all GKB-containing media, and the corresponding control and 6-OHDA groups received an equivalent amount of DMSO. For the protection experiments, cells were pretreated with GKB for 2 h; 6-OHDA was then added directly to the same medium at a final concentration of 200 µM, and incubation was continued for an additional 24 h. GKB remained present throughout the 6-OHDA exposure. The untreated control, vehicle control, GKB-only, 6-OHDA-only, and GKB + 6-OHDA cultures were subjected to identical medium volumes and incubation periods. To examine the effects of GKB alone on cellular metabolic activity, cells were treated with GKB at 5, 10, 20, 30, or 40 µg/mL for 24 h. For the concentration-response protection experiments, cells were pretreated with the same GKB concentrations for 2 h, then exposed to 200 µM 6-OHDA for 24 h without GKB removal. The concentration used in the subsequent experiments was selected according to two prespecified criteria: it should not reduce CCK-8-derived metabolic activity when applied alone and should provide the greatest preservation of metabolic activity in 6-OHDA-treated cells. Based on these criteria and the post hoc comparisons among the tested concentrations, 20 µg/mL GKB, corresponding to approximately 47.1 µM, was selected for the subsequent experiments. Following completion of the treatments, 10 µL of CCK-8 reagent was added to each well in 100 µL of culture medium. Plates were incubated at 37 °C in the dark for 2 h. Absorbance was measured at 450 nm using a Multiskan™ SkyHigh microplate spectrophotometer (Thermo Fisher Scientific, Waltham, MA, USA). Absorbance values obtained from wells without cells but containing culture medium and CCK-8 reagent were subtracted as background. CCK-8-derived metabolic activity was expressed as a percentage relative to the untreated control group. A single biological value was created by averaging the values obtained from the technical wells in each biological replicate.

*Following dose selection, the experimental groups were formed as follows:*

Control group: Cells cultured under standard conditions without any active treatment.

GKB group: Cells treated with 20 µg/mL GKB.

6-OHDA group: Cells exposed to 200 µM 6-OHDA for 24 h.

GKB + 6-OHDA group: Cells pre-incubated with 20 µg/mL GKB for 2 h, followed by exposure to 200 µM 6-OHDA for 24 h.

The control and single-treatment groups were subjected to the same total experimental duration as the combined treatment group. Each experiment was performed with six biological replicates obtained from independent cell cultures on different days (*n* = 6). Technical wells in the same biological replicate were not considered as independent samples.

### 2.3. Biochemical Analyses

Intracellular reduced glutathione (GSH), malondialdehyde (MDA), superoxide dismutase (SOD), poly(ADP-ribose) polymerase-1 (PARP-1), and caspase-3 levels were measured according to the manufacturer’s instructions using commercial assay kits (Abbkine, China). The total protein concentration of each sample was determined by the Bradford method. GSH, MDA, SOD, PARP-1, and caspase-3 results were normalised to total protein amount to reduce variability due to cell density. Results were expressed as ng/mg protein, nmol/mg protein, U/mg protein, or as a percentage of the control, respectively. All absorbance measurements were performed using a Multiskan™ SkyHigh microplate spectrophotometer. A standard curve was generated for each parameter, and only sample values falling within the measurement range were included in the analysis.

### 2.4. PI/Hoechst Double Staining

Cell death was assessed using PI/Hoechst 33342 double staining. SH-SY5Y cells were seeded into glass-bottomed culture dishes and washed with PBS after experimental procedures. Cells were incubated in a staining solution containing 2 µg/mL PI and 4 µM Hoechst 33342 at room temperature in the dark for 25 min. Fluorescence images were obtained using an Axiovert 5 fluorescence microscope (Zeiss, Oberkochen, Germany) and ZEN Blue software (version 3.12, Zeiss). 535/617 nm and 350/461 nm excitation/emission filters were used for PI and Hoechst, respectively. Objective magnification, exposure time, light intensity, camera gain, and other imaging settings were kept constant across all groups. At least five non-overlapping microscopic fields were imaged from each biological sample using a predetermined systematic-random sampling method. Field selection was performed using the Hoechst or bright-field channel before visualising the signal in the PI channel. The percentage of PI-positive cells was calculated as the number of PI-positive cells divided by the total number of Hoechst-positive nuclei in the same field. Images were analysed by a researcher blinded to group information using the same threshold values. The mean of the fields from the same biological sample was used as a single value in the statistical analysis.

### 2.5. Intracellular ROS Analysis

Intracellular oxidant-sensitive fluorescence was assessed using DCFH-DA. Following application, cells were washed with serum-free culture medium and incubated in medium containing 3 µM DCFH-DA at 37 °C in the dark for 30 min. Cells were washed three times with PBS to remove excess extracellular probe. DCF fluorescence was imaged using an Axiovert 5 fluorescence microscope at approximately 488 nm excitation and 525 nm emission wavelengths. The same objective, exposure time, light intensity, and camera settings were used in all experimental groups. At least five non-overlapping areas were evaluated from each biological sample. After subtracting background fluorescence, the corrected average fluorescence intensity per cell was calculated using ZEN Blue software (ZEN Blue 3.12). A comparable number of cells were analysed between groups. The average area from the same sample was considered a single biological value, and results were presented as a percentage of the control group. Because DCFH-DA is not specific to any particular ROS, the results were interpreted as general intracellular oxidant-sensitive fluorescence rather than specifically H_2_O_2_ or superoxide levels.

### 2.6. Intracellular Ca^2+^ Imaging

Intracellular Ca^2+^ responses were evaluated using the Ca^2+^-sensitive probe Fluo-4 AM. Following the experimental treatments, cells were washed with serum-free medium and incubated with 1 µM Fluo-4 AM at 37 °C in the dark for 30 min. Cells were washed three times with PBS and incubated in dye-free medium at room temperature for 15 min to allow intracellular de-esterification. Baseline fluorescence was recorded before sequential application of 1 mM H_2_O_2_ and 25 µM ACA. ACA was used to assess the ACA-sensitive component of the H_2_O_2_-evoked response; because ACA is not selective for TRPM2, its effect was not interpreted as direct evidence of TRPM2 activity. The same fields were followed throughout baseline, H_2_O_2_, and ACA measurements. Individual cells were delineated as regions of interest using identical segmentation criteria. Mean cellular fluorescence was corrected by subtracting the signal measured in a cell-free region of the same field (F^c^ = Fcell − Fbackground). Corrected values were normalised to the mean baseline fluorescence of the vehicle-control cultures and expressed as a percentage, with the vehicle-control baseline defined as 100%. Measurements from cells and fields within each independently prepared culture were averaged to obtain one biological value at each measurement stage. Cells and fields were treated as subsamples, whereas six independently prepared cultures performed on different days constituted the biological replicates (*n* = 6). Illumination, exposure time, detector gain, acquisition settings, and analysis parameters were kept constant, and image analysis was performed blinded to treatment group.

### 2.7. Statistical Analysis

**Figure 1:**
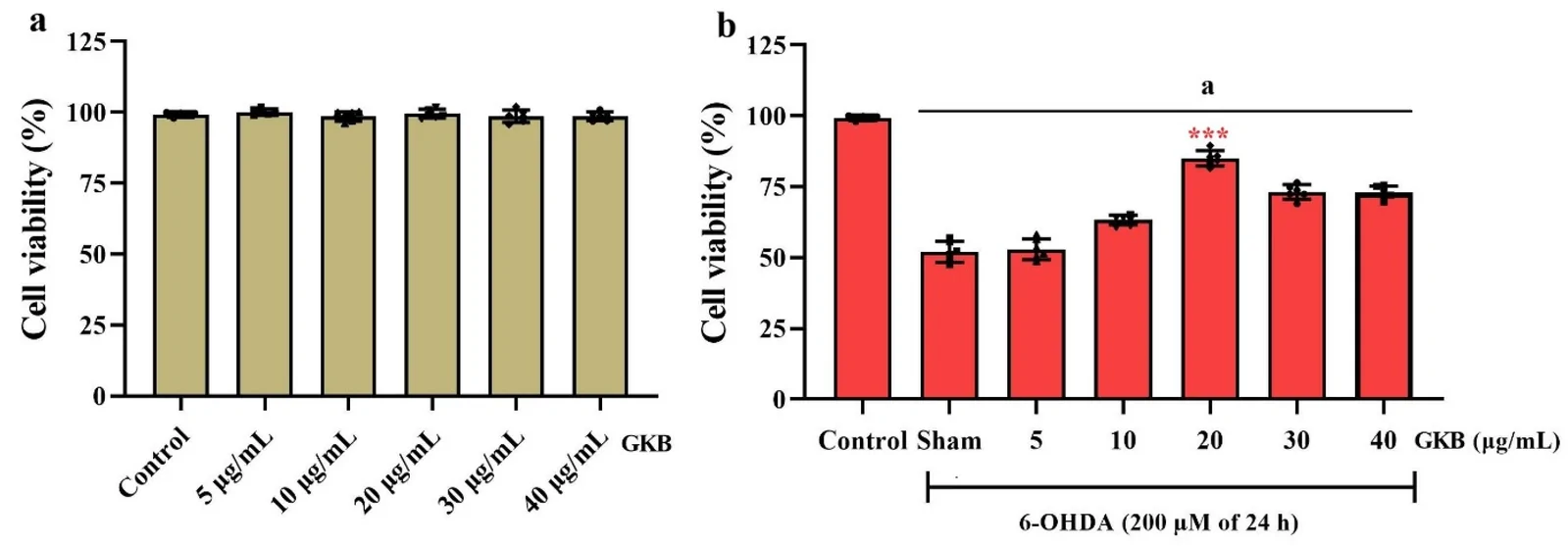
Effects of GKB on CCK-8-derived metabolic activity in SH-SY5Y cells exposed to 6-OHDA. (**a**) Effects of GKB alone (5–40 µg/mL, 24 h) on cellular metabolic activity. (**b**) Effects of GKB pretreatment (5–40 µg/mL, 2 h) on cellular metabolic activity following exposure to 6-OHDA (200 µM, 24 h). GKB remained in the medium during 6-OHDA exposure. Cellular metabolic activity was evaluated using the CCK-8 assay and expressed as a percentage of the untreated control. Data are presented as mean ± SD from six independent biological replicates (*n* = 6), with technical wells averaged to obtain one value per replicate. GKB-only groups were compared with the untreated control using one-way ANOVA followed by Dunnett’s test. The 6-OHDA and 6-OHDA + GKB groups were analysed using one-way ANOVA followed by Tukey’s multiple-comparisons test. Among the concentrations tested, 20 µg/mL GKB preserved CCK-8-derived metabolic activity the most, whereas increasing the concentration beyond 20 µg/mL provided no additional benefit. ^a^ *p* < 0.001 compared with the untreated control; *** *p* < 0.001 for 20 µg/mL GKB + 6-OHDA compared with each of the other GKB + 6-OHDA dose groups.

**Figure 2:**
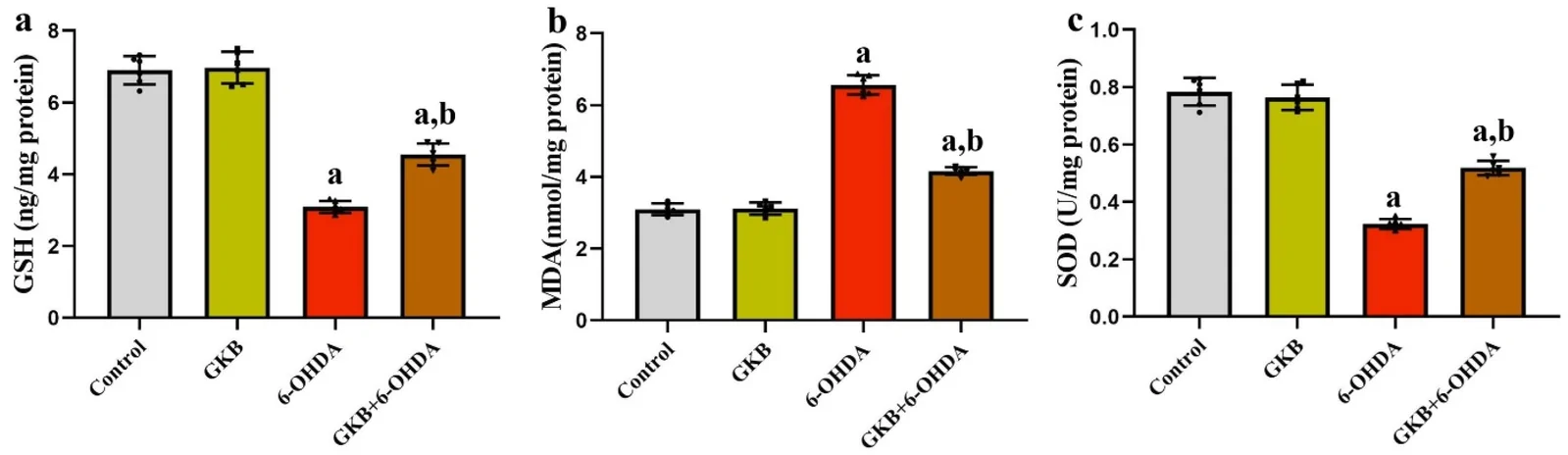
Effects of GKB on 6-OHDA-induced changes in oxidative-stress parameters. (**a**) GSH, (**b**) MDA, and (**c**) SOD levels in SH-SY5Y cells. Data are presented as mean ± SD from six independently prepared cell cultures performed on different days (*n* = 6). Data were analysed using one-way ANOVA followed by Tukey’s multiple-comparisons test. Horizontal brackets indicate the post hoc comparisons presented in the graphs. ^a^ *p* < 0.001 compared with the vehicle-control group; ^b^ *p* < 0.001 compared with the 6-OHDA group.

**Figure 3:**
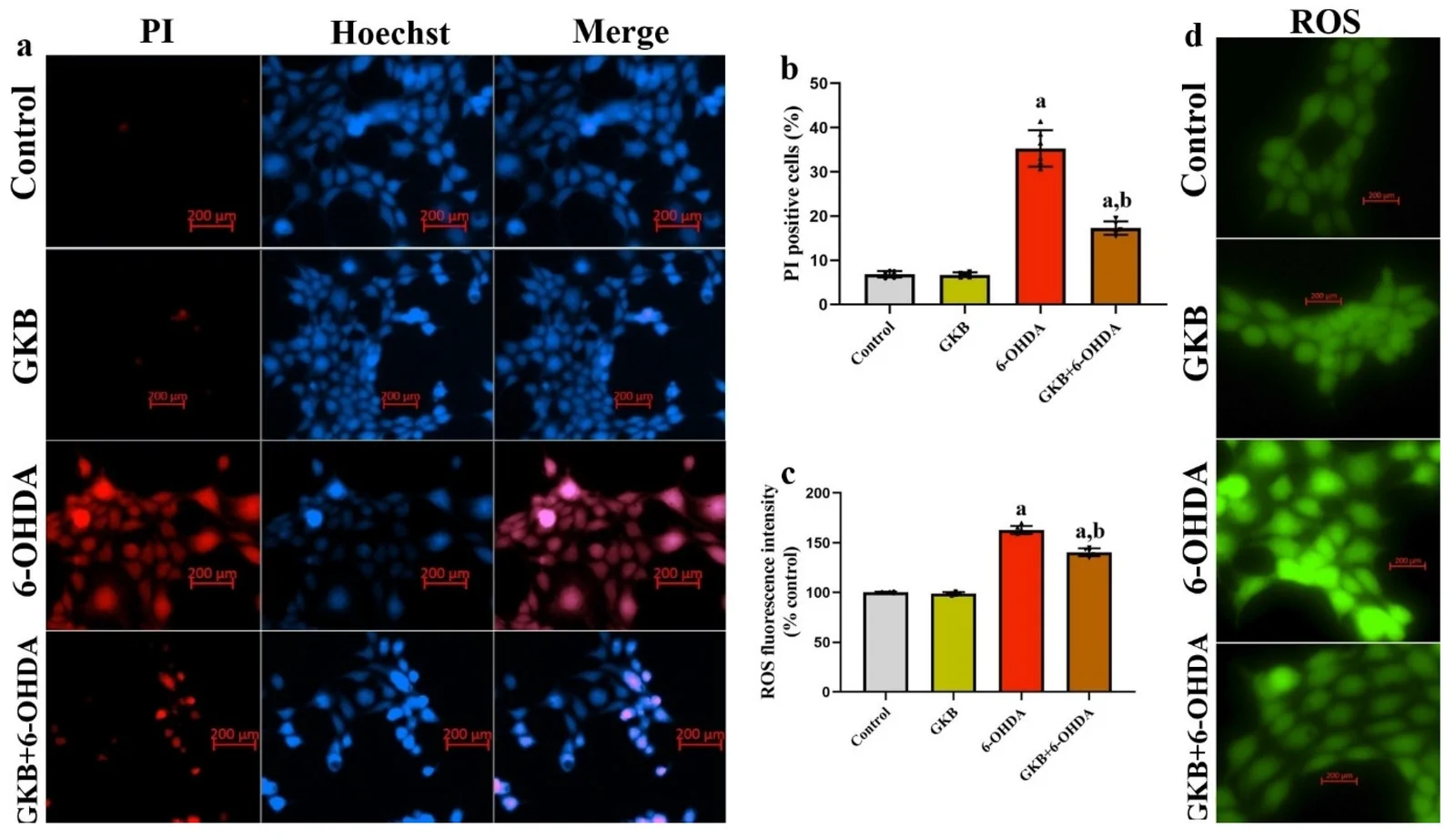
Effects of GKB on 6-OHDA-induced loss of membrane integrity and intracellular oxidant-sensitive fluorescence. (**a**) Representative images of PI/Hoechst 33342 double staining; (**b**) percentage of PI-positive cells relative to the total number of Hoechst-positive nuclei; (**c**) quantitative analysis of background-corrected DCF fluorescence; and (**d**) representative DCFH-DA fluorescence images. For each biological replicate, at least five non-overlapping fields were selected using a systematic-random sampling procedure. Measurements from fields within the same independently prepared culture were averaged to obtain a single biological value. Thus, at least 30 microscopic fields were analysed per experimental group across six independent cultures (*n* = 6), and individual fields or cells were not treated as independent experimental units. DCF fluorescence was quantified as the mean cellular fluorescence intensity after subtraction of the background signal measured in a cell-free region of the same field and was expressed relative to the vehicle-control group. Data are presented as mean ± SD and were analysed using one-way ANOVA followed by Tukey’s multiple-comparisons test. Horizontal brackets indicate the post hoc comparisons presented in the graphs. ^a^ *p* < 0.001 compared with the vehicle-control group; ^b^ *p* < 0.001 compared with the 6-OHDA group. Scale bars: 200 µm in all representative fluorescence images.

**Figure 4:**
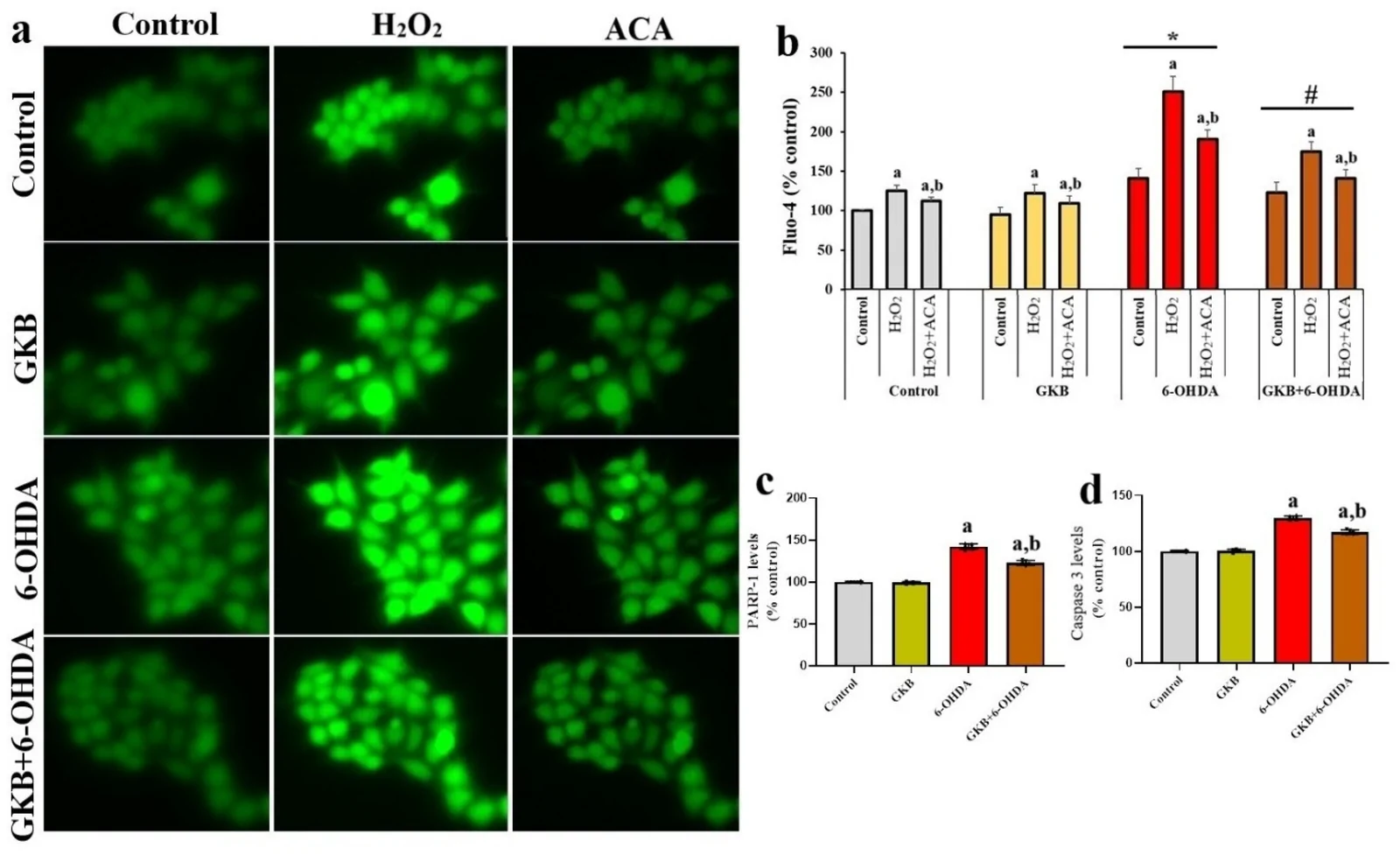
Effects of GKB on the oxidant-evoked Fluo-4 response and total PARP-1 and caspase-3 levels in 6-OHDA-treated cells. (**a**) Representative Fluo-4 fluorescence images obtained at baseline and following sequential H_2_O_2_ and ACA application; (**b**) quantitative analysis of normalised Fluo-4 fluorescence; (**c**) total PARP-1 levels; and (**d**) total caspase-3 levels. In each biological replicate, the same microscopic fields and cells were followed during baseline, H_2_O_2_ stimulation, and subsequent ACA treatment. For each cell, mean fluorescence intensity was corrected by subtracting the background signal measured in a cell-free region of the same field. The background-corrected fluorescence values were normalised to the mean baseline fluorescence of the vehicle-control cultures, which was defined as 100%. Measurements from individual cells and fields within each independently prepared culture were averaged to obtain a single biological value per measurement condition. The independent culture, rather than the individual cell or field, was considered the experimental unit (*n* = 6). Fluo-4 data were analysed using two-way repeated-measures ANOVA with the Geisser–Greenhouse correction, followed by Šídák’s multiple-comparisons test. Total PARP-1 and caspase-3 data were analysed using one-way ANOVA followed by Tukey’s multiple-comparisons test. Horizontal brackets indicate the specific post hoc comparisons. The PARP-1 and caspase-3 assays measured total protein abundance and did not distinguish cleaved or enzymatically active forms. ^a^ *p* < 0.001 compared with the corresponding baseline; ^b^ *p* < 0.001 compared with the corresponding H_2_O_2_-stimulated value; * *p* < 0.001 compared with the H_2_O_2_-stimulated vehicle-control group; # *p* < 0.001 compared with the H_2_O_2_-stimulated 6-OHDA group. For panels (**c**,**d**), ^a^ *p* < 0.001 compared with the vehicle-control group and ^b^ *p* < 0.001 compared with the 6-OHDA group.

Statistical analyses were performed using GraphPad Prism software (version 8.2; GraphPad Software, Boston, MA, USA). Data are presented as mean ± standard deviation (SD) from six independently prepared cell cultures performed on different days (*n* = 6). Technical wells, individual cells, and microscopic fields from the same independently prepared culture were treated as subsamples and averaged to obtain a single biological value per endpoint. For each analysis, the independent culture was considered the experimental unit. Normality and homogeneity of variance were assessed using the Shapiro–Wilk and Levene’s tests, respectively. In the GKB-only concentration experiment shown in Figure 1a, groups were analysed using one-way ANOVA followed by Dunnett’s multiple-comparisons test, with each GKB concentration compared with the vehicle-control group. The 6-OHDA concentration-response experiment shown in Figure 1b and the endpoints shown in Figure 2, Figure 3 and Figure 4c,d were analysed using one-way ANOVA followed by Tukey’s multiple-comparisons test. Sequential Fluo-4 measurements obtained from the same cultures under baseline, H_2_O_2_-stimulated, and ACA-treated conditions were analysed using two-way repeated-measures ANOVA, with treatment group as the between-subject factor and measurement condition as the within-subject factor. The Geisser–Greenhouse correction was applied, followed by Šídák’s multiple-comparisons test. Adjusted *p*-values are reported, and *p* < 0.05 was considered statistically significant. For descriptive reporting, percentage changes were calculated from the group means relative to the specified reference group as [(mean of the comparison group − mean of the reference group)/mean of the reference group] × 100. These percentage changes were not used for hypothesis testing.

## 3. Results

### 3.1. GKB Preserved CCK-8-Derived Metabolic Activity in 6-OHDA-Treated SH-SY5Y Cells

GKB applied alone at concentrations of 5–40 µg/mL did not significantly alter CCK-8-derived metabolic activity compared with the untreated control (Dunnett-adjusted *p* > 0.05 for all comparisons; Figure 1a). Exposure to 200 µM 6-OHDA reduced metabolic activity by approximately 48% relative to the control group (*p* < 0.001; Figure 1b). GKB pretreatment produced a non-monotonic concentration–response pattern. Although the other concentrations yielded smaller numerical increases, 20 µg/mL GKB increased metabolic activity by approximately 64% relative to 6-OHDA alone and produced significantly higher values than the other GKB + 6-OHDA concentration groups (Tukey-adjusted *p* < 0.001 for each comparison). Because 20 µg/mL GKB, equivalent to approximately 47.1 µM, did not impair metabolic activity when administered alone and produced the greatest protective response during 6-OHDA exposure, it was selected for the subsequent experiments.

### 3.2. GKB Reduced 6-OHDA-Induced Oxidative Stress

Relative to the control group, 6-OHDA reduced GSH and SOD levels by approximately 56% and 59%, respectively, and increased MDA by approximately 115% (*p* < 0.001 for each comparison; Figure 2a–c). GKB alone did not significantly alter these parameters. Compared with 6-OHDA alone, GKB pretreatment increased GSH by approximately 48% and SOD by 62%, while reducing MDA by 37% (*p* < 0.001 for each comparison). Nevertheless, GSH and SOD remained approximately 34% below their corresponding control values, whereas MDA remained approximately 35% above the control value, indicating partial rather than complete recovery.

### 3.3. GKB Reduced 6-OHDA-Induced Loss of Membrane Integrity and Intracellular Oxidant-Sensitive Fluorescence

PI/Hoechst staining showed that 6-OHDA increased the proportion of PI-positive cells from approximately 7% in the control group to 35%, corresponding to an absolute increase of 28 percentage points (*p* < 0.001; Figure 3a,b). GKB pretreatment reduced PI positivity to approximately 17%, representing an absolute reduction of 18 percentage points and a relative reduction of 51% compared with 6-OHDA alone (*p* < 0.001). Intracellular DCF fluorescence increased by approximately 64% following 6-OHDA exposure (*p* < 0.001; Figure 3c,d). GKB pretreatment reduced this signal by approximately 14% relative to 6-OHDA alone (*p* < 0.001), although it remained approximately 41% above the control value. Because DCFH-DA does not identify a specific reactive species or its cellular source, these results are reported as changes in intracellular oxidant-sensitive fluorescence rather than ROS production.

### 3.4. GKB Attenuated the Oxidant-Evoked Ca^2+^ Response and Changes in Total PARP-1 and Caspase-3 Levels

H_2_O_2_ increased Fluo-4 fluorescence by approximately 25%, 29%, 78%, and 42% relative to the corresponding baseline values in the control, GKB, 6-OHDA, and GKB + 6-OHDA groups, respectively (*p* < 0.001 for each comparison; Figure 4a,b). Under H_2_O_2_-stimulated conditions, the Fluo-4 signal in the 6-OHDA group was approximately 102% higher than that in the control group (*p* < 0.001). GKB pretreatment reduced the enhanced response by approximately 31% compared with 6-OHDA alone (*p* < 0.001). Subsequent ACA administration reduced the H_2_O_2_-stimulated signal by approximately 10%, 11%, 24%, and 20% in the control, GKB, 6-OHDA, and GKB + 6-OHDA groups, respectively (*p* < 0.001), although the values did not fully return to their corresponding baselines. In the biochemical analyses, 6-OHDA increased total PARP-1 and total caspase-3 levels by approximately 42% and 31%, respectively, relative to the control (*p* < 0.001; Figure 4c,d). GKB pretreatment reduced these increases by approximately 14% and 10%, respectively, compared with 6-OHDA alone (*p* < 0.001). Because the assays did not distinguish cleaved or enzymatically active forms, these changes were not interpreted as evidence of apoptosis.

## 4. Discussion

In this study, the effect of GKB on 6-OHDA-induced SH-SY5Y cell damage was evaluated through CCK-8-derived metabolic activity, oxidative stress, loss of membrane integrity, total PARP-1 and caspase-3 levels, and intracellular Ca^2+^ responses. 6-OHDA application decreased CCK-8-derived metabolic activity and GSH and SOD levels, while increasing MDA levels, oxidant-sensitive DCF fluorescence, the PI-positive cell ratio, total PARP-1 and total caspase-3 levels. Additionally, the H_2_O_2_-stimulated Ca^2+^ response was enhanced in 6-OHDA-treated cells and reduced by ACA application. GKB pre-treatment significantly mitigated the changes induced by 6-OHDA. The findings suggest that the protective effect of GKB may be related to its support of redox balance, its limitation of cell death, and attenuation of the enhanced oxidant-stimulated Ca^2+^ response. 6-OHDA is a neurotoxin commonly used to study dopaminergic neuronal damage. Intracellular uptake of 6-OHDA via catecholamine transporters leads to the formation of ROS and reactive quinone products through rapid auto-oxidation. This process is associated with impaired mitochondrial respiration, weakening of antioxidant defences, and activation of cell death pathways [7,8]. It has been shown previously that 6-OHDA treatment of SH-SY5Y cells reduces cell viability, increases ROS and lipid peroxidation, and enhances mitochondrial apoptotic signalling [19,20,21]. In this study, the significant reduction in CCK-8-derived metabolic activity following exposure to 200 µM 6-OHDA provided a sufficient experimental range for evaluating the protective effect of GKB.

The 6-OHDA concentration used causes acute and significant cellular damage. This feature allows comparison of the short-term effects of potential protective compounds, but it differs from the chronic, progressive pathology of PD. Therefore, the findings were evaluated in the context of acute oxidative cell damage induced by 6-OHDA, rather than the entirety of clinical PD. The tumour origin of SH-SY5Y cells, their lack of neuron–glia interactions, and their limited representation of senescence-related cellular characteristics also determine the model’s biological scope [9,22]. Application of GKB alone at concentrations of 5–40 µg/mL did not significantly alter CCK-8-derived metabolic activity. Although the CCK-8 assay alone cannot exclude all forms of cytotoxicity, these findings indicate that the tested concentrations did not impair cellular metabolic activity under the experimental conditions used. In the protective concentration screening, GKB produced a non-monotonic response, with the greatest preservation of CCK-8-derived metabolic activity observed at 20 µg/mL. Based on its molecular weight of 424.4 g/mol, this concentration corresponds to approximately 47.1 µM and is close to the 40 µM GKB concentration previously used in bupivacaine-treated SH-SY5Y cells [23]. Non-monotonic neuroprotective responses have also been reported for other compounds. Epigallocatechin-3-gallate produced a bell-shaped protective response in SH-SY5Y cells, with its greatest effect occurring within an intermediate concentration range [24]. Similarly, the BDNF loop-4 mimetic GSB-106 showed a bell-shaped survival response in serum-deprived SH-SY5Y cells [25]. These examples indicate that increasing the concentration of a neuroactive compound does not necessarily result in progressively greater protection. In the present study, lower GKB concentrations may have been insufficient to produce a detectable response, whereas concentrations above 20 µg/mL provided no additional benefit. Because the screening was limited to CCK-8-derived metabolic activity, the biological basis of this pattern remains undetermined. Therefore, 20 µg/mL should be regarded as the empirically optimal concentration under the present experimental conditions rather than a universally effective dose.

The neuroprotective effects of GKB have been previously reported in different cellular damage models. It has been shown that pre-treatment of GKB in bupivacaine-treated SH-SY5Y cells increased cell viability and reduced ROS accumulation, mitochondrial depolarization, endoplasmic reticulum stress, and cell death [23,24,26]. GKB has been reported to reduce lipid, protein, and nucleic acid oxidation and support GSH and SOD-related antioxidant responses in undifferentiated and differentiated SH-SY5Y cells treated with amyloid-β_1–42_ [18]. It has also been observed in different experimental models that ginkgolides can protect mitochondrial function and regulate oxidative stress-related cell death signals [27]. The CCK-8-derived metabolic activity findings are consistent with the reported protective properties of GKB against different neurotoxic stimuli.

In oxidative stress analyses, 6-OHDA application decreased GSH and SOD levels and increased MDA and DCF fluorescence. GSH plays a key role in the detoxification of reactive electrophilic products and the removal of cellular peroxides. SOD, on the other hand, contributes to the limitation of radical chain reactions by enabling the conversion of superoxide radical to hydrogen peroxide. Deficiencies in these systems can increase the oxidation of polyunsaturated membrane lipids and MDA formation [3,28]. The GSH, SOD, and MDA findings in this study are consistent with previous studies reporting that 6-OHDA weakens antioxidant defence and increases lipid peroxidation in SH-SY5Y cells [29,30].

Chen et al. reported that 6-OHDA application reduced GSH synthesis and cell viability in SH-SY5Y cells, while strengthening antioxidant defence mitigated cell loss [29]. Kim et al. showed that 6-OHDA-induced ROS formation and apoptosis can be reduced by supporting the Nrf2/ARE-mediated antioxidant response [31]. Similarly, administration of astragaloside-IV reduces the increase in 6-OHDA-induced MDA and ROS [30]. These findings support the idea that maintaining antioxidant capacity may be a key cytoprotective approach against 6-OHDA-induced damage.

The fact that GKB pre-treatment increased GSH and SOD levels and decreased MDA and oxidant-sensitive fluorescence suggests that the protective effect may be related to its support of cellular redox balance. GKB may have limited the depletion of endogenous antioxidant defences; however, the present measurements do not establish which reactive species or cellular sources contributed to the DCF signal. Although GKB has been reported to support mitochondrial function in other models [32,33]. The fact that some values in the GKB + 6-OHDA group did not fully return to control levels suggests that GKB provided partial protection rather than completely eliminating 6-OHDA-induced damage.

The DCFH-DA assay detected treatment-related changes in intracellular probe oxidation but did not identify the reactive species responsible for the signal or their subcellular source. This probe is not specific to any particular type of ROS, and its oxidation can be affected by cellular peroxidases, metal ions, and different reactive intermediates [34]. Therefore, the change in DCF fluorescence has been interpreted as a general change in cellular oxidant load rather than a specific H_2_O_2_ or superoxide concentration. The convergence of DCFH-DA results with GSH, SOD, and MDA findings provides complementary evidence supporting the effect of GKB on redox balance. Using analytical methods based on MitoSOX, electron paramagnetic resonance, or specific oxidation products to investigate ROS sources may contribute to a more detailed evaluation of this mechanism.

PI/Hoechst double-staining showed that 6-OHDA increased the proportion of cells that had lost membrane integrity, whereas GKB pre-treatment reduced this increase. Taken together, the CCK-8 and PI/Hoechst findings indicate that GKB preserved metabolic activity and reduced the proportion of cells with compromised plasma-membrane integrity under the present experimental conditions. These measurements do not identify the specific mode of cell death. Decreased mitochondrial membrane potential following 6-OHDA exposure has been associated with cytochrome-c release, disruption of the Bax/Bcl-2 balance, caspase activation, and PARP-1-related changes [31,35,36]. These processes were not measured here and therefore cannot be inferred from the present PI/Hoechst or ELISA findings. Accordingly, neither the PI/Hoechst findings nor the ELISA measurements permit classification of the observed cell death as apoptosis. Such a conclusion would require complementary evidence, including Annexin V-based analysis, caspase activity measurements, or detection of cleaved caspase-3 and cleaved PARP-1.

In the biochemical analyses, 6-OHDA increased total PARP-1 and total caspase-3 levels, whereas GKB attenuated these changes. PARP-1 participates in the cellular response to DNA damage, while caspase-3 is synthesised as an inactive procaspase that requires proteolytic processing to acquire apoptotic activity. However, changes in the total abundance of these proteins do not indicate enzymatic activation or apoptosis. Accordingly, the present findings should be interpreted only as treatment-related changes in total PARP-1 and caspase-3 levels. PARP-1 is activated in response to DNA strand breaks, and its limited activation contributes to DNA repair. Conversely, prolonged or excessive PARP-1 activity can contribute to NAD^+^ and ATP depletion, disruption of cellular energy balance, and cell death [37,38]. Caspase-3, on the other hand, is one of the key executive proteases of mitochondrial and death receptor-mediated apoptotic pathways. It has been reported that PARP-1 and caspase-3-related responses are increased in experimental Parkinsonism models, and that protective interventions can reduce these changes [31,35,36].

The reduction in total PARP-1 and caspase-3 levels following GKB treatment was accompanied by reduced oxidant-sensitive fluorescence and fewer PI-positive cells. The reduction in ROS formation may have limited DNA damage and the associated PARP-1 response. The parallel changes in PI positivity, oxidant-sensitive fluorescence, and total PARP-1 and caspase-3 abundance indicate treatment-related cellular injury, but they do not establish a specific molecular cell-death pathway [29,39]. Since the biochemical analyses measured total protein levels, the findings were interpreted only as changes in total protein abundance, not as evidence of enzymatic activation or apoptosis, or as direct evidence of enzymatic activation of apoptosis. Adding Annexin V/PI flow cytometry, along with analyses of cleaved caspase-3 and cleaved PARP-1, could provide a more detailed elucidation of GKB’s effects on cell death pathways.

One striking finding of the study is the significant increase in H_2_O_2_-stimulated Fluo-4 fluorescence in cells treated with 6-OHDA. There is a bidirectional interaction between oxidative stress and intracellular Ca^2+^ homeostasis. While ROS can activate redox-sensitive Ca^2+^ channels, increased cytosolic Ca^2+^ can also increase mitochondrial metabolic load and ROS production [10,11,40,41]. This cycle may contribute to the conversion of initial oxidative stimulation into more persistent cellular damage.

TRPM2 is a Ca^2+^-permeable cation channel regulated by oxidative stress, intracellular Ca^2+^, and ADP-ribose. Previous studies have implicated TRPM2 in oxidant-induced neuronal damage and shown that increased TRPM2 expression enhances the susceptibility of SH-SY5Y cells to oxidative injury [42,43]. However, TRPM2 expression and channel activity were not directly assessed in the present study. Moreover, ACA is not selective for TRPM2 and can inhibit other TRP channels. Therefore, the ACA-mediated reduction in Fluo-4 fluorescence indicates only the presence of an ACA-sensitive component of the oxidant-evoked Ca^2+^ response and cannot be considered evidence of TRPM2-dependent Ca^2+^ entry. Importantly, the lower H_2_O_2_-evoked Fluo-4 response observed after GKB pretreatment should not be interpreted as evidence that GKB directly inhibits TRPM2 or another Ca^2+^ channel. GKB also reduced oxidant-sensitive fluorescence and MDA levels while preserving GSH and SOD, indicating a reduction in the cellular oxidative burden. A more plausible explanation is therefore that the lower Ca^2+^ response occurred secondarily to the antioxidant effect of GKB, through reduced stimulation of redox-sensitive Ca^2+^-entry pathways and improved maintenance of intracellular Ca^2+^ homeostasis. Effects on other plasma-membrane channels, intracellular Ca^2+^ stores, or Ca^2+^-extrusion mechanisms cannot be excluded. The present experiments cannot distinguish these indirect effects from a direct action of GKB on a specific ion channel.

The study examined several distinct aspects of 6-OHDA-induced cellular injury using metabolic, biochemical, and fluorescence-based measurements. However, because each endpoint was assessed using a single analytical approach, agreement among these related findings should not be regarded as method-independent validation of any individual measurement. PI/Hoechst staining indicated loss of membrane integrity, while the PARP-1 and caspase-3 assays showed treatment-related changes in the total abundance of these proteins. The investigation of the effect of GKB alone and at different concentrations contributed to the experimental determination of the appropriate working dose. Separating biological replicates from technical measurements also enhanced the interpretability of the data.

Several limitations should be considered. The study was conducted in a single undifferentiated SH-SY5Y cell line, which does not reproduce primary dopaminergic neurons, neuron–glia interactions, aging-related changes, or the chronic progression of Parkinson’s disease [44]. In addition, GKB was administered only before acute 6-OHDA exposure; Therefore, the findings represent a preventive in vitro paradigm rather than therapeutic efficacy after established injury. TRPM2 expression, ADP-ribose generation, and channel currents were not assessed, and because ACA is not selective for TRPM2, the ACA-sensitive Ca^2+^ response cannot be attributed specifically to TRPM2, nor can the present data distinguish an indirect antioxidant effect of GKB from a direct action on Ca^2+^ transport. Similarly, PI/Hoechst staining indicates a loss of plasma membrane integrity but does not identify the mode of cell death, whereas total PARP-1 and caspase-3 measurements do not demonstrate cleavage, enzymatic activation, or apoptosis. Furthermore, the quantitative findings for individual endpoints were not cross-validated on an independent analytical platform: ELISA-based measurements, including total PARP-1 and caspase-3 levels, were not confirmed by Western blotting, and fluorescence microscopy-based measurements were not verified by flow cytometry. This lack of orthogonal validation reduces the robustness of the quantitative conclusions and limits confidence in the precise magnitude of the reported effects. Likewise, DCFH-DA fluorescence reflects intracellular probe oxidation but cannot identify a specific reactive species or its cellular source. Finally, mitochondrial membrane potential, respiration, ATP production, and mitochondrial oxidant formation were not measured; Therefore, the findings do not establish that GKB protects mitochondria.

## 5. Conclusions

GKB pretreatment attenuated the 6-OHDA-induced reduction in CCK-8-derived metabolic activity and partially preserved GSH and SOD levels while reducing MDA, PI positivity, intracellular oxidant-sensitive fluorescence, and the increases in total PARP-1 and caspase-3 abundance in undifferentiated SH-SY5Y cells. GKB also moderated the enhanced H_2_O_2_-evoked Fluo-4 response. The reduction produced by ACA indicates an ACA-sensitive component of this response; however, it does not demonstrate TRPM2-dependent Ca^2+^ entry, as ACA is not selective and TRPM2 expression and channel activity were not assessed. Likewise, the present findings do not establish apoptosis, mitochondrial protection, or the production of a specific ROS. These questions require complementary cell death assays, direct measurements of mitochondrial function, species-specific oxidant analyses, and target-specific molecular or electrophysiological approaches.

## Data Availability

The raw data supporting the conclusions of this article will be made available by the authors on request.

## Abbreviations

6-OHDA	6-Hydroxydopamine
ACA	N-(p-amylcinnamoyl)anthranilic acid
ADP-ribose	Adenosine diphosphate ribose
ANOVA	Analysis of variance
ATCC	American Type Culture Collection
Ca^2+^	Calcium ion
CCK-8	Cell Counting Kit-8
DCF	2′,7′-Dichlorofluorescein
DCFH-DA	2′,7′-Dichlorodihydrofluorescein diacetate
DMEM/F-12	Dulbecco’s Modified Eagle Medium/Ham’s F-12
DMSO	Dimethyl sulfoxide
FBS	Fetal bovine serum
GKB	Ginkgolide B
GSH	Reduced glutathione
H_2_O_2_	Hydrogen peroxide
MDA	Malondialdehyde
PARP-1	Poly(ADP-ribose) polymerase-1
PBS	Phosphate-buffered saline
PD	Parkinson’s disease
PI	Propidium iodide
ROS	Reactive oxygen species
SD	Standard deviation
SOD	Superoxide dismutase
TRPM2	Transient receptor potential melastatin 2
